# *Trypanosoma cruzi* High Mobility Group B (*Tc*HMGB) can act as an inflammatory mediator on mammalian cells

**DOI:** 10.1371/journal.pntd.0005350

**Published:** 2017-02-08

**Authors:** Pamela Cribb, Virginia Perdomo, Victoria L. Alonso, Romina Manarin, Jorge Barrios-Payán, Brenda Marquina-Castillo, Luis Tavernelli, Rogelio Hernández-Pando

**Affiliations:** 1 Instituto de Biología Molecular y Celular de Rosario (IBR), CONICET, Rosario, Argentina; 2 Facultad de Ciencias Bioquímicas y Farmacéuticas, Universidad Nacional de Rosario (UNR), Rosario, Argentina; 3 Sección de Patología Experimental, Instituto Nacional de Ciencias Médicas y Nutrición Salvador Zubirán, INCMNSZ, Mexico City, Mexico; Instituto de Ciências Biológicas, Universidade Federal de Minas Gerais, BRAZIL

## Abstract

**Background:**

High Mobility Group B (HMGB) proteins are nuclear architectural factors involved in chromatin remodeling and important nuclear events. HMGBs also play key roles outside the cell acting as alarmins or Damage-associated Molecular Patterns (DAMPs). In response to a danger signal these proteins act as immune mediators in the extracellular milieu. Moreover, these molecules play a central role in the pathogenesis of many autoimmune and both infectious and sterile inflammatory chronic diseases.

**Principal findings:**

We have previously identified a High mobility group B protein from *Trypanosoma cruzi* (*Tc*HMGB) and showed that it has architectural properties interacting with DNA like HMGBs from other eukaryotes. Here we show that TcHMGB can be translocated to the cytoplasm and secreted out of the parasite, a process that seems to be stimulated by acetylation. We report that recombinant *Tc*HMGB is able to induce an inflammatory response *in vitro* and *in vivo*, evidenced by the production of Nitric Oxide and induction of inflammatory cytokines like TNF-α, IL-1β and IFN-γ gene expression. Also, TGF-β and IL-10, which are not inflammatory cytokines but do play key roles in Chagas disease, were induced by r*Tc*HMGB.

**Conclusions:**

These preliminary results suggest that *Tc*HMGB can act as an exogenous immune mediator that may be important for both the control of parasite replication as the pathogenesis of Chagas disease and can be envisioned as a pathogen associated molecular pattern (PAMP) partially overlapping in function with the host DAMPs.

## Author summary

When an infection occurs, the innate immune cells recognize Pathogen Associated Molecular Patterns (PAMPs) through their Pattern Recognition Receptors. This triggers an inflammatory response intended to kill the foreign microbe. But inflammation can also be triggered by the recognition of endogenous molecules called “Danger (or Damage) Associated Molecular Patterns” (DAMPs) that are released by damaged or necrotic cells to “ring the alarm” of the immune system that repair is needed, so some of them are also known as “alarmins”. High Mobility group box 1 protein (HMGB1) is a prototypical alarmin molecule released by injured cells and it is also actively secreted by cells of the innate immune system in response to invasion as well as to sterile damage. *Trypanosoma cruzi*, the causal agent of Chagas Disease, has its own HMGB protein that we called *Tc*HMGB. Using *in vitro* and *in vivo* experimental systems, we demonstrated for the first time that *Tc*HMGB is able to mediate inflammation on mammalian cells, inducing the expression of both pro-inflammatory and anti-inflammatory cytokines. Our results suggest that the parasite´s protein could have a role in the immune response and the pathogenesis of Chagas disease, probably overlapping to some extent with the host cell DAMP molecules´ functions.

## Introduction

Chagas disease is considered by the World Health Organization (WHO) as one of the neglected tropical diseases (NTD) causing illness and hampering economic development in poor populations. It is caused by the protozoan parasite *Trypanosoma cruzi* and, like other infectious diseases, it can be fatal. According to the latest reports of the WHO, it is estimated that nearly 6 to 7 million people are infected by *T*. *cruzi* worldwide. Most cases occur in Latin America where Chagas disease is endemic and the parasite is mainly transmitted by an insect vector. However, as a consequence of human migrations the distribution of many illnesses has been changing in the last decades and Chagas disease has been increasingly detected in the United States of America, Canada, and many European and Western Pacific countries (http://www.who.int/mediacentre/factsheets/fs340/en/). Humans usually acquire the infection when a triatomine insect releases metacyclic trypomastigotes with its feces after a blood meal. Metacyclic trypomastigotes can pass through the damaged skin or intact mucosa and, once inside the body, infect a variety of cells. Other routes of infection have been also described: oral, congenital, via blood transfusion or by organ transplantation. Inside the host cell, metacyclic trypomastigotes transform into amastigotes, the intracellular replicative form found in vertebrate hosts. After several cycles of binary division, amastigotes differentiate back to trypomastigotes which are released upon cellular lysis, invading nearby nucleated cells and being disseminated through the bloodstream to other organs and tissues. In most people, the infection has a self-limiting acute phase, which is usually asymptomatic. During this stage, parasites replicate into the cytoplasm of a variety of cell types including macrophages, muscle cells, adipocytes and cells of the central nervous system and they can be found in blood and tissues in high numbers [1,2]. This acute phase lasts about 50–60 days and is characterized by high parasitaemia and tissue parasitism and a strong activation of the innate immunity with the concomitant high plasma levels of inflammatory cytokines like Tumor Necrosis Factor-α (TNF-α), interleukin (IL)-12 and interferon-γ (IFN-γ) as well as nitrogen reactive intermediates. Also during the acute phase, B- and T- cell are activated leading to the establishment of the adaptive immune response. The immune response usually controls the *T*. *cruzi* infection but fails in the complete eradication of the parasite, so people remain infected for life establishing a dynamic equilibrium with the parasite in the chronic phase of the disease, where parasitaemia and tissue parasitism are very low [3,4]. Most chronic infected individuals remain asymptomatic, but some of them develop different complications after a decade or more [5]. About 20% to 30% of patients will experience chronic Chagasic myocarditis with sequelae including heart failure, arrhythmias, thromboembolism and eventually death. Another 15% to 20% will experience chronic digestive sequelae like megaesophagus and megacolon [6]. Is not yet fully understood why different patients develop different clinical forms of the disease ranging from asymptomatic to severe cardiac problems. Also it is noteworthy the high inflammatory response associated to a relative low parasite number during the chronic phase leading to the suggestion of an autoimmune component in the disease pathogenesis. Many questions are still unsolved, but it is well known that both parasite and host response to infection contribute to the pathogenesis of Chagas disease [7].

High Mobility Group B proteins (HMGBs) are highly abundant proteins that play important biological roles both inside and outside the cell. HMGBs are nuclear DNA binding proteins involved in chromatin remodeling and they are key players in the control of transcription, DNA replication, recombination and DNA repair [8,9]. Besides the nuclear functions of all HMGBs, HMGB1 of humans and other mammals has been largely studied because it is a well-recognized Damage Associated Molecular Pattern (DAMP) molecule that is secreted by immune cells or released by injured cells “alarming” the immune system to trigger an inflammatory response [10–12]. It has been implicated in the pathogenesis of several inflammatory disorders like sepsis, atherosclerosis and arthritis and also autoimmune diseases like systemic lupus erythematosus [13].

Proteins belonging to the HMGB family have been identified in wide range of organisms from yeast to human including several protozoan and helminth parasites [14–18]. We have previously characterized the DNA-binding functions of *Trypanosoma cruzi* HMGB (*Tc*HMGB) suggesting it may be an important player in transcription control in this parasite [18]. In this report we evaluated the ability of *Tc*HMGB to induce cytokine production in a first attempt to study its putative role as an immune-mediator in the pathogenesis of Chagas disease. Recombinant *Tc*HMGB (r*Tc*HMGB) was able to induce an inflammatory response *in vitro* and *in vivo*, evidenced by the production of Nitric Oxide and the induction of inflammatory cytokines like TNF-α, IL-1β and IFN-γ gene expression. Interestingly, Transforming growth factor-β (TGF-β) and IL-10, usually associated to the opposite effect (anti-inflammatory) and known to play key roles in chronic chagasic myocardiopathy, were also induced by r*Tc*HMGB. Moreover, experimental *T*. *cruzi* infection in BALB/c mice, showed during acute infection numerous cardiomyocytes and macrophages heavily infected with amastigotes, which showed strong TcHMGB immunostaining. TcHMGB immunostaining was also observed in fibrin located intravascular and attached to the endocardium in coexistence with numerous inflammatory cells that showed strong immunostaining to TNF-α, IL-1β and IFN-γ. Thus, immunohistochemistry in mice hearts during acute experimental *T*. *cruzi* infection showed high production of TcHMGB by amastigotes that apparently is secreted and co-exist with inflammatory cells that are producing pro-inflammatory cytokines.

These results suggest that *Tc*HMGB can act as an exogenous immune mediator that may be important for the pathogenesis of Chagas disease and can be envisioned as a pathogen associated molecular pattern (PAMP) partially overlapping in function with the host DAMPs.

## Methods

### Ethics statement

All the animal work was done according to the guidelines of the Mexican constitution law NOM 062-200-1999, and approval of the Ethical Committee for Experimentation in Animals of the National Institute of Medical Sciences and Nutrition in Mexico (CINVA) (PAT1021), and all efforts were made to minimize suffering. All experiments were approved by the Institutional Animal Care and Use Committee of the School of Biochemical and Pharmaceutical Sciences, National University of Rosario (Argentina) (File 6060/227) and conducted according to specifications of the US National Institutes of Health guidelines for the care and use of laboratory animals.

### Expression and purification of recombinant TcHMGB suitable for immunological assays

Recombinant proteins expression in *Escherichia coli* and affinity-chromatography purification, particularly Glutathione S transferase (GST) fusion *Tc*HMGB (r*Tc*HMGB) and GST (rGST), have been previously optimized in our laboratory [18]. However, since the recombinant protein was expressed in *E*. *coli*, we had to rule out the possibility of endotoxin or lipopolysaccharide (LPS) contamination, which could mask the putative effect of *Tc*HMGB as a mediator of inflammation. To achieve our purpose, we modified the previous protocol adding a Polymixin B incubation step, to bind contaminant endotoxin previous to r*Tc*HMGB purification. The whole bacterial lysate was passed through a Polymixin B-affinity chromatography column (Detoxi-Gel Endotoxin Removing Gel, Thermo Scientific, Argentina) obtaining a “detoxyfied lysate”. Then, the r*Tc*HMGB GST-fusion protein was purified with a Glutathione-agarose specific affinity chromatography column (Sigma Aldrich, Argentina). Recombinant GST was purified following the same protocol and this protein was used as negative control in the immune assays. The purity of the detoxyfied recombinant protein was determined by SDS–PAGE and Pierce LAL Chromogenic Endotoxin Quantitation Kit (Thermo Scientific, Argentina). Protein concentration was quantified using Pierce BCA Protein Assay kit (Thermo Scientific).

### Rabbit polyclonal antibodies production

Rabbits were inoculated with recombinant TcHMGB protein to raise antibodies. Formal animal ethics approval was given for this work by the School of Biochemistry of Rosario National University, Argentina, Ethics Committee. Animals were housed and maintained according to the institution’s experimental guidelines for animal studies. Specific antibodies against the parasite HMGB protein were affinity purified from the antiserum by using recombinant proteins immobilized on an agarose matrix. Briefly, after binding the protein to the matrix, it was cross-linked with 1% formaldehyde in PBS for 15 min and extensively washed with at least 50 vol. of PBS. The antiserum was then passed three times through the column containing the immobilized protein and extensively washed with PBS to separate non-specific antibodies. Finally, anti-TcHMGB-specific antibodies were eluted with 0.1 M Triethylamine (pH 11.5) and immediately neutralized to pH 8 with HCl. Purified antibodies were then concentrated through filtration and conserved in 50% glycerol at -20°C. Purified antibody specificity was tested using immunoblotting assays.

### Slot blot of culture supernatants

Supernatants were obtained after 6 h of incubation in RPMI medium without FCS from: *T*. *cruzi* epimastigotes, trypomastigotes and infected Vero cells. Uninfected Vero cell cultures and recombinant TcHMGB protein were used as negative and positive controls respectively. At the beginning of the incubation period deacetylase inhibitors were added to half of the samples, Nicotinamide 100 μM (Sigma Aldrich), Sodium Butyrate 5 mM (Sigma Aldrich) and Trichostatin A 1 μM (Sigma Aldrich).

The supernatants were concentrated 20 times by ultrafiltration and 20 μl from each sample was blotted onto nitrocellulose filters in a slot blot devise (BioRad) following the manufacturer´s instructions. The blotted proteins were visualized with Ponceau S staining. The membranes were treated with 5% non-fat milk in PBS for 1 h and then incubated with specific antibodies diluted in PBS for 3 hs. The antibodies used were affinity-purified polyclonal rabbit anti-TcHMGB, monoclonal mouse anti-trypanosome a-tubulin clone TAT-1 (a gift from K. Gull, University of Oxford, UK) and rabbit polyclonal anti-SAPA (a gift from Dr. Oscar Campetella, IIB-INTECH, Argentina). Bound antibodies were detected using peroxidase-labelled anti-mouse or anti-rabbit IgGs (GE Healthcare, Buckinhamshire, UK) and ECL Prime (GE Healthcare) according to the manufacturer’s protocol.

### Immunocytolocalization

Trypomastigotes and exponentially growing epimastigotes were incubated with deacetylase inhibitors Nicotinamide 100 μM (Sigma Aldrich), Sodium Butyrate 5 mM (Sigma Aldrich) and Trichostatin A 1 μM (Sigma Aldrich) for 6 hs. Then they were centrifuged, washed twice with PBS, settled on polylysine-coated coverslips, and fixed with 4% paraformaldehyde in PBS at room temperature for 20 min. The fixed parasites were washed with PBS and permeabilized with 0.2% Triton X-100 in PBS for 10 min. After washing with PBS, the parasites were incubated with rabbit anti-TcHMGB primary antibody diluted in 1% BSA in PBS for 3 h at room temperature. Nonbound antibodies were washed with 0.01% Tween 20 in PBS, and then the slides were incubated with fluorescence-conjugated anti-rabbit IgG (fluorescein; Jackson ImmunoResearch) and 2 μg ml^-1^ DAPI (4,6-diamidino-2-phenylindole) for 1 h. The slides were washed with 0.01% Tween 20 in PBS and finally mounted with VectaShield (Vector Laboratories). Images were acquired with a confocal Nikon Eclipse TE-2000-E2 microscope using Nikon EZ-C1 software or an epifluorescence Nikon Eclipse Ni-U microscope. Adobe Photoshop CS and ImageJ software were used to pseudocolor and process all images.

### Cell culture and treatments

The murine macrophage cell line RAW 264.7 (ATCC TIB-71) was cultured in Dulbecco's Modified Eagle's Medium (DMEM, Invitrogen, Carlsbad, CA, USA), supplemented with 10% (v/v) Fetal Calf Serum (FCS, Natocor, Córdoba, Argentina) and 1% (v/v) of a mixture of antibiotics (10,000 units/ml penicillin and 10,000 g/ml streptomycin, Sigma, Argentina) in a humidified atmosphere containing 5% CO_2_ at 37°C.

Cells were then seeded in 24-well plates at a density of 4.5 × 10^5^ cells/well and maintained in culture for 24 h in DMEM supplemented with 2% (v/v) FCS and 1% (v/v) antibiotics mixture. After 24 h culture, the medium was renewed with the addition of r*Tc*HMGB (10 μg/ml), rGST (10 μg/ml) or LPS (100 ng/ml) in the corresponding wells. Only DMEM (supplemented with FCS and antibiotics) was added to non-treated control cells. After 3, 6 or 24 h culture supernatants were collected for Nitric Oxide (NO) production determination (see below). Cells were washed once in sterile Phosphate-buffered saline (PBS) and then collected with Versene Solution (Thermo Fisher Scientific, Life Technologies, Argentina) and transferred to a clean tube for RNA purification.

### Nitric Oxide production determination

NO produced by RAW cells was determined by evaluating the nitrite content in the culture supernatants with the Griess reagent, a flourometric reagent used for the quantitative analysis of nitrite in solution [19]. Absorbance at 543 nm was determined, and NO concentration was calculated by comparison with Abs_543nm_ of standard curve of NaNO_2_ solutions prepared in culture medium.

### MTT viability assay

Cell viability after all the treatments was determined by the 3-(4,5-dimethylthiazol-2-yl)-2,5-diphenyltretazolium bromide (MTT) reduction assay. Briefly, RAW cells were incubated in 96 well plate in the presence of the different treatments (r*Tc*HMGB, rGST, LPS or DMEM alone) for 24 h. Then 20 μl MTT solution (5mg/mL in PBS) were added to each well and incubated for 1 h at 37°C. After this incubation period, MTT solution was removed and precipitated formazan was solubilized in 200 μl dimethyl sulfoxide (DMSO). Optical density (OD) was quantified spectrophotometrically (measurement λ = 530 nm, reference λ = 630 nm) using a microplate reader LD-400 (Beckman Coulter, Brea, CA, USA). DMSO was used as blank. Each treatment was performed in triplicate.

### Animal treatments

Male BALB/c mice, 7 weeks old, were obtained from our own breeding facilities. Animals were feed *ad libitum* with a standard laboratory pellet diet and were allowed free access to water during treatment. Mice were randomly divided into four experimental groups. The different treatments were administered intraperitoneally (i.p.) in a unique dose in sterile saline solution as vehicle (r*Tc*HMGB 1μg, rGST 50 μg, LPS 1μg), control group only received the vehicle. Groups of four animals were euthanized by ex-sanguination and under anesthesia with pentobarbital at 3, 6 and 24 h after the injection, the spleens were immediately removed, frozen in liquid nitrogen and maintained under -70°C until RNA extraction.

In order to detect by immunohistochemistry rTcHMGB and cytokines in cardiac lesions produced by *T*. *cruzi* infections, BALB/c mice were subcutaneously injected with 1000 viable trypomastigotes of the Tulahuen strain of *T*. *cruzi*. Animals had access to food and water ad libitum and were allocated in temperature-controlled rooms under light–dark 12-h cycles and handled according to institutional guidelines. After 14 (acute) or 100 (chronic) days of infection, mice were killed and hearts were removed and sliced transversally into three sections. Tissue samples were fixed in buffered formalin or embedded in Tissue-Tek (Miles Inc., Elkhart, USA) and frozen in liquid nitrogen.

### Immunohistochemistry

For the histological study, the heart samples from infected mice were fixed by immersion in 10% formaldehyde dissolved in PBS. Transversal heart sections were dehydrated and embedded in paraffin (Oxford Labware, St Louis, MO, USA), sectioned and stained with hematoxylin and eosin (HE). The local expression of TcHMGB and the cytokines TNFα, IFN-γ, IL-1 and IL-10 was determined by immunohistochemistry (IHC). Tissue sections 5 μm-width were obtained and placed on slides loaded with poly l-lysine (Biocare Medical, Lake Concord, CA, USA). For dewaxing, the slides were placed at 60–70°C for 20 min and incubated for 5 min into xylene. The slides were changed five times into the medium in the following sequence: (i) xylene–alcohol (1:1), (ii) absolute alcohol, (iii) alcohol 96%, and (iv) distilled H_2_O. Once hydrated, endogenous peroxidase was blocked with methanol–10% H_2_O_2_. The washings were performed with HEPES-buffered saline (HBS)–Tween 20 (10 mM HEPES, 150 mM NaCl, 2 mM CaCl_2_, 0.05% Tween 20). The areas of tissue were delineated and then blocked with 100 μl of HBS with 2% Background Sniper (Biocare Medical), and incubated for 30 min in a humid chamber. Then the sections were incubated with appropriate dilutions of rabbit polyclonal specific antibodies to TcHMGB, TNF-α, IFN-γ, IL-1 and IL-10 (Santa Cruz Biotechnology, Santa Cruz, CA, USA) overnight at room temperature. Subsequently, slides were washed and 100 μl of goat anti-rabbit antibody-horseradish peroxidase (AB/HRP) (Vectastain ABC System, Burlingame, CA, USA) was added and incubated for 30 min to be revealed with 100 μl of diaminobenzidine/ H_2_O_2_ (0.004 g diaminobenzidine + 10 mL HBS + 4 mL H_2_O_2_). Slides were washed and contrasted with hematoxylin.

### RNA extraction and Quantitative Real Time Reverse Transcription PCR (qRT-PCR)

After treatment, total RNA was isolated from RAW 264.7 cells or mouse spleens using TRI Reagent RT (Molecular Research Center Inc., Cincinnati, USA) according to manufacturer's instructions. Quality and quantity of RNA were evaluated through agarose gel electrophoresis and spectrophotometry (Abs_260nm/280nm_), respectively. Samples were treated with RQ1 RNase-free DNase (Promega, Wisconsin, USA) to remove possible DNA contamination prior to Reverse Transcription (RT). Five micrograms of RNA were used in the RT reaction with oligo (dT) and the Omniscript kit (Qiagen, Inc.) according to manufacturer's instructions to obtain the cDNA. Real time PCR reactions were carried out on a StepOne (Applied Biosystem, ThermoFisher Scientific, Foster city, CA, USA) with Power SYBR Green PCR Master Mix (Invitrogen, Carlsbad, CA, USA) using specific primers (Table 1).

Results for cytokine mRNAs were normalized to the 60S acidic ribosomal protein P0 (RPLP0) mRNA as housekeeping gene, based on the standard curve quantitative method [20]. The specificity of each reaction was verified with a melting curve between 55°C and 95°C with continuous fluorescence measure.

**Table 1:** Primers used for the analysis of cytokine mRNA expression by qRT-PCR.

Gene	Gene Description	Accession number	Primer sequence	Annealing Temp (°C)
RPLP0	RPLP0 ribosomal protein	NM_007475.5	Fw 5ʹ-CTCTCGCTTTCTGGAGGGTG-3´	60
			Rv 5ʹ-ACGCGCTTGTACCCATTGAT-3´	
TNF-α	Tumor necrosis factor α	AK014374	Fw 5ʹ-TGTGGCTTCGACCTCTACCTC-3ʹ	60
			Rv 5ʹ-GCCGAGAAAGGCTGCTTG-3ʹ	
IL-1β	Interleukin 1β	NM_008361	Fw 5´- TTGACGGACCCCAAAAGATG-3´	55
			Rv 5´-AGAAGGTGCTCATGTCCTCA-3´	
INF-γ	Interferon γ	NM_008337	Fw 5ʹ-GGTGACATGAAAATCCTGCAG-3ʹ	60
			Rv 5ʹ-CTCAAACTTGGCAATACTCATGA-3ʹ	
TGF-β	Transforming growth factor β	NM_011577	Fw 5ʹ-GCTGATCCCGTTGATTTCCA-3ʹ	60
			Rv 5ʹ-GTGGCTGAACCAAGGAGACG-3ʹ	
IL-10	Interleukin 10	NM_010548.2	Fw 5´-GGTTGCCAAGCCTTATCGGA-3´	62
			Rv 5´-ACCTGCTCCACTGCCTTGCT-3´	

### Obtaining standards to absolute quantification of qRT-PCR

For analytical evaluation of the real-time RT-PCR, gene specific PCR amplicons of each gen were prepared as standards, PCR products were analyzed by 1% agarose gel electrophoresis and purified (GFX PCR DNA and gel band Purification kit; GE Healthcare life sciences, Pittsburgh, PA, USA), cloned into pCR2.1TOPO (Invitrogen, Carlsbad, CA, USA) and sequenced. Concentration of the purified nucleic acid was calculated by measuring the absorbance at 260 nm and its corresponding concentration was converted into copies per microlitre by using the Avogadro constant (6.023 x 10^23^) and its molecular weight (number of bases of the PCR product plus number of bases of vector, multiplied by the average molecular weight of a pair of nucleic acids). Tenfold serial dilutions of the quantified construct solutions were kept in aliquots at -20°C and used throughout the study as external standards of known concentration for the real-time PCR reaction (range of the standards: 10^3^–10^6^ copies/μl). The calibration curve was created by plotting the threshold cycle (Ct) corresponding to each standard, versus the value of their corresponding log number of each gen concentration (expressed as copies/μl).

### Statistical analysis

Statistical analysis was performed with GraphPad Prism 3.0 software (GraphPad Software, La Jolla, CA, USA) using the One-Way Analysis of variance (ANOVA) followed by Dunnett´s post-hoc test. Data are expressed as the fold change of the control group, presented as mean ± standard error of the mean (SEM). Significance was set at p<0.05.

## Results

### Protein acetylation stimulates TcHMGB relocalization to the cytoplasm and shedding

**Fig 1:**
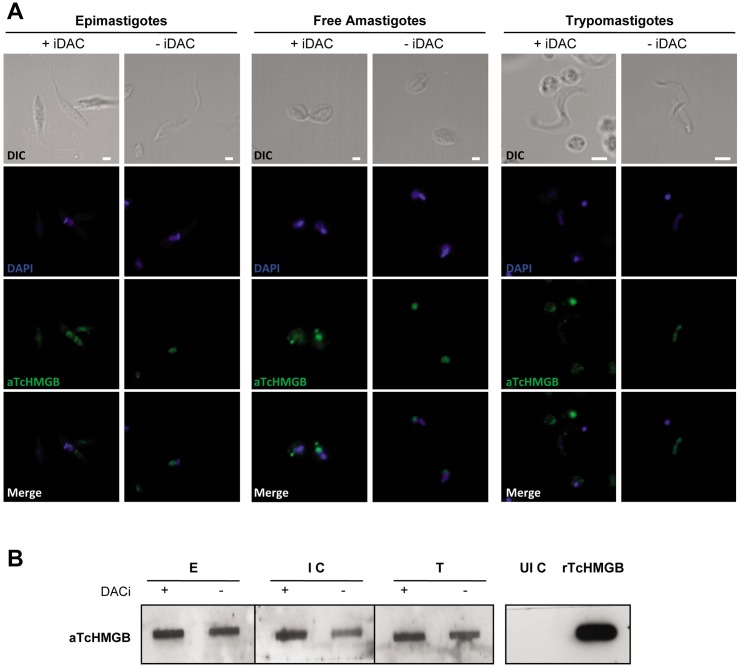
Acetylation promotes TcHMGB re-localization from the nucleus to the cytoplasm and shedding to the extracellular medium. (A) Immunofluorescence assay using purified anti-TcHMGB antibody and parasites at different stages of the *T*. *cruzi* life cycle: Epimastigotes, Free Amastigotes released from infected Vero cells and Trypomastigotes treated (+) and non-treated (-) with deacetylase inhibitors (iDAC) Trichostatin A, Sodium Butyrate and Nicotinamide. Anti-rabbit IgG conjugated to fluorescein was used as a secondary antibody. Nuclei and kinetoplasts were labeled with DAPI. Bars = 2 μm. (B) Slot western blot assay. Culture supernatants in FCS-free media were treated (+) and non-treated (-) with deacetylase inhibitors (iDAC) and blotted onto nitrocellulose membranes. Epimastigotes: E, Infected Vero cells: I C, Trypomastigotes: T, and un-infected Vero cells: UI C. Rabbit anti-TcHMGB (aTcHMGB) antibodies were used to detect the presence of TcHMGB. Recombinant TcHMGB (rTcHMGB) was used as positive control.

Under normal conditions, TcHMGB is located in the nucleus in all *T*. *cruzi* life cycle stages [18]. Like other HMGB family members, TcHMGB lacks a leader sequence that would enable its secretion by the classical route, so we decided to investigate if acetylation can promote its redirection to the cytoplasm and eventual secretion, as already described for mammalian and Schistosoma HMGB proteins [21,22]. We treated the parasites with a combination of deacetylase inhibitors (DACis), namely sodium butyrate, Trichostatin A and Nicotinamide, which target different deacetylases, and then analyzed the effect over protein localization on epimastigote and trypomastigote forms of the parasite by immunofluorescence with specific anti-TcHMGB antibodies. As can be seen in Fig 1A, we observed a relocalization of TcHMGB from the nucleus to the cytoplasm upon DACi treatment, suggesting that acetylation promotes TcHMGB translocation to the cytoplasm.

In order to determine if TcHMGB can be secreted extracellularly, we tested the presence of TcHMGB in supernatants from *T*. *cruzi*-infected Vero cells as well as from trypomastigotes and epimastigotes cell-free media. Also, taking into account the relocalization observed after DACi treatment (Fig 1A), we performed the secretion assay comparing DACi-treated and non-treated parasites or infected cells. We observed an immunoreactive band corresponding to TcHMGB in epimastigote, trypomastigote and infected cells supernatants, but not in uninfected Vero cells supernatant (Fig 1B). As expected, the DACi treated samples, show a more intense band, consistent with the acetylation-induced secretion hypothesis. However, the presence of TcHMGB in the supernatants of non-treated parasites and infected cells suggests that acetylation may stimulate TcHMGB secretion, but it is probably not the only mechanism. Anti-tubulin gave no signal when incubated with the same supernatants, demonstrating that the TcHMGB immunoreactive band is due to the presence of the protein in the extracellular medium and not a consequence of parasite lysis. Also, the shed acute phase antigen (SAPA) was detected in the trypomastigote and infected cells supernatants, confirming the presence of a well-known secreted protein in the extracellular samples (S1 Fig) [23].

### Recombinant *Tc*HMGB can act as an inflammatory mediator in cultured RAW cells

In order to analyze if *Tc*HMGB has pro-inflammatory properties like other HMGB family members, *in vitro* cultured RAW 264.7 cells were treated with r*Tc*HMGB for 3, 6 and 24 hours. Nitric Oxide production by RAW cells was determined by quantification of nitrite in the culture supernatants by the Griess reaction, which is usually used as an estimation of NO production as a consequence of macrophage activation [19]. Also, cells were collected and the expression of different cytokines was determined by quantitative RT-PCR (qRT-PCR). LPS-treatment was used as pro-inflammatory control whereas, as negative controls, we used both non treated cells and recombinant GST-treatment. None of the treatments affected cellular viability as determined by MTT reduction assay (S1 Table).

**Fig 2:**
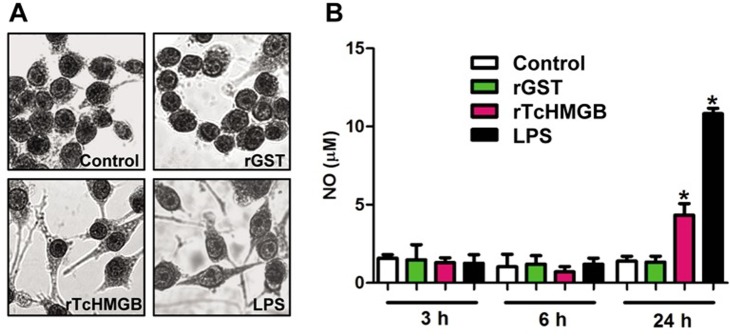
RAW 264.7 macrophages are activated and produce Nitric oxide after rTcHMGB-stimulation. (A) Representative microscopy of macrophages after indicated treatments. (B) NO production at 3, 6, 24 h after the indicated treatments estimated by Griess reaction by comparison to NaNO_2_ standards. The experiment was performed in triplicates and the bar graphs represent the mean values ± SEM; * p<0.05.

In general, poor effect was noted at shorter times (3 and 6 hours), but at 24 hours post-treatment, RAW cells treated with r*Tc*HMGB and with LPS were clearly activated, showing the typical “star-like” activated macrophage morphology (Fig 2A) and an increase in the NO production compared to negative controls (Fig 2B).

**Fig 3:**
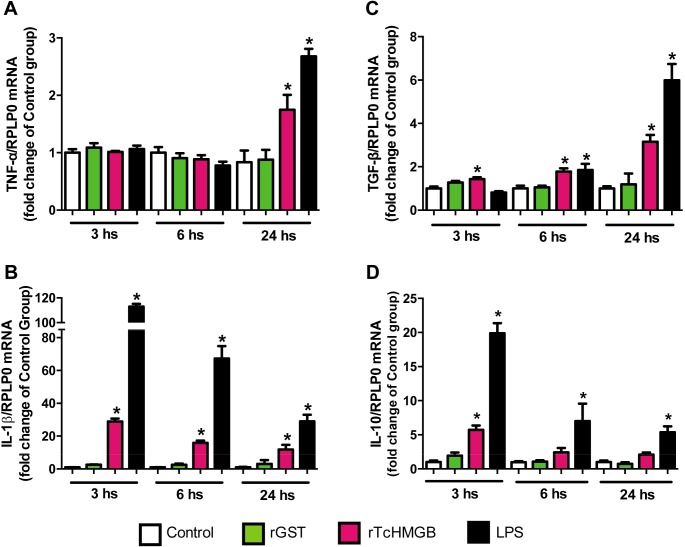
Pro- and anti-inflammatory cytokines´ expression was induced on cultured RAW cells after rTcHMGB treatment. Relative cytokines´ mRNA levels at 3, 6 and 24 h of rTcHMGB, rGST or LPS treatment were determined by qRT-PCR using RPLP0 as housekeeping gene. Data are expressed as the fold change of the control non-treated group (white column) and presented as Mean ± SEM; * p<0.05. Cytokines analyzed were (A) TNF-α, (B) IL-1β, (C) TGF-β, (D) IL-10.

Recombinant *Tc*HMGB stimulation also induced TNF-α and IL-1β expression (Fig 3A and 3B respectively), to a lesser extent than LPS-treatment but higher than non-treated or GST-treated cells. TNF-α expression was detected on macrophages only at 24 hours post-induction with r*Tc*HMGB but not at shorter times. In contrast, IL-1β expression was triggered much earlier. At 3 hours of r*Tc*HMGB-treatment, we observed an important increase in IL-1β expression that slowly decreases as treatment time elapses, but staying above the negative controls expression levels during the whole duration of the experiment.

Notably, also gene expression of TGF-β and IL-10, which are normally associated to an anti-inflammatory effect, increased after r*Tc*HMGB treatment (Fig 3C and 3D). TGF-β expression showed to be slightly higher in r*Tc*HMGB-treated cells than in control cells at 3 h post-treatment and continued increasing with time reaching the maximum at 24 h. On the other hand, IL-10 expression was raised at 3h but decreased at longer times.

Notably, for all the cytokines analyzed, the expression pattern was similar after TcHMGB- and LPS-treatment, although in most cases, LPS induced more radical changes. This data show that, like LPS, *Tc*HMGB can act as an inflammatory mediator *in vitro*.

### *Tc*HMGB can induce cytokine production *in vivo* and may have a role in the pathogenesis of Chagas disease

**Fig 4:**
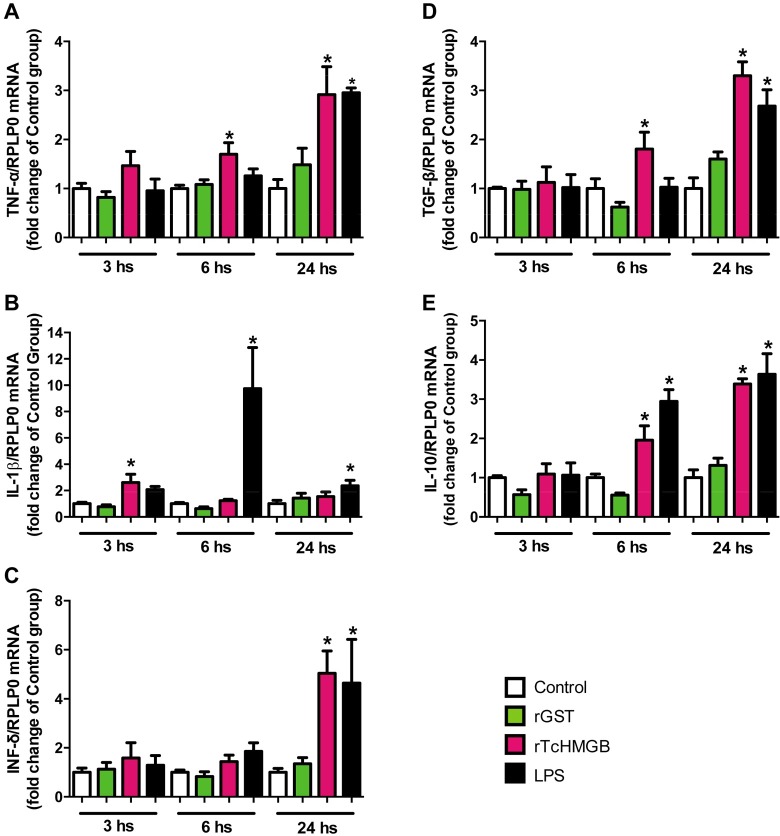
Pro- and anti-inflammatory cytokines´ expression was induced after mice treatment with rTcHMGB. Total RNA was extracted from mice spleens at 3, 6 and 24 h post inoculation with rTcHMGB, rGST, LPS or vehicle (Physiological solution), and relative cytokines´ mRNA levels were determined by qRT-PCR using RPLP0 as housekeeping gene. Data are expressed as the fold change of the control non-treated group (white column) and presented as Mean ± SEM; * p<0.05. Cytokines analyzed were (A) TNF-α, (B) IL-1β, (C) IFN-γ, (D) TGF-β, (E) IL-10.

The activity of *Tc*HMGB as a potential mediator of the immune response was also tested in an *in vivo* system. We performed a similar experiment treating BALB/c mice with r*Tc*HMGB and pro- and anti-inflammatory cytokines expression was evaluated by qRT-PCR from spleen samples at 3, 6 or 24 hours post-inoculation (p.i.). Interestingly, in this *in vivo* test we obtained similar results to the previous *in vitro* assays (Fig 4). Both TNF-α and IL-1β were induced after treatment with r*Tc*HMGB. TNF-α induction was significantly higher in r*Tc*HMGB-treated mice at 6 hours p.i., and continued increasing for at least 24 hours (Fig 4A). Like in the *in vitro* assay, IL-1β expression was induced in r*Tc*HMGB-treated mice earlier than TNF-α, being significantly higher than the negative controls at 3 hours p.i., but then decreasing to reach levels similar to those of negative controls (Fig 4B). In this *in vivo* system, we also evaluated the expression of IFN-γ, which is a key cytokine in the pathogenesis of Chagas disease (but it is not expressed by macrophages). As can be seen in Fig 4C, IFN-γ expression clearly increased at 24 h p.i., reaching levels comparable to those of LPS-treatment.

As well as in the *in vitro* assay, mice treated with r*Tc*HMGB showed increased TGF-β and IL-10 expression (Fig 4D and 4E). In this case, both TGF-β and IL-10 show a statistically significant difference from controls at 6 h, that become greater at 24 h p.i.

These results suggest that *Tc*HMGB not only can induce an inflammatory response on the host, but also it may participate in the consequent control of inflammation, which can be related to the parasite need to persist in the host.

### TcHMGB released from amastigotes in infected tissues can be related to inflammatory cytokines production during acute infection

**Fig 5:**
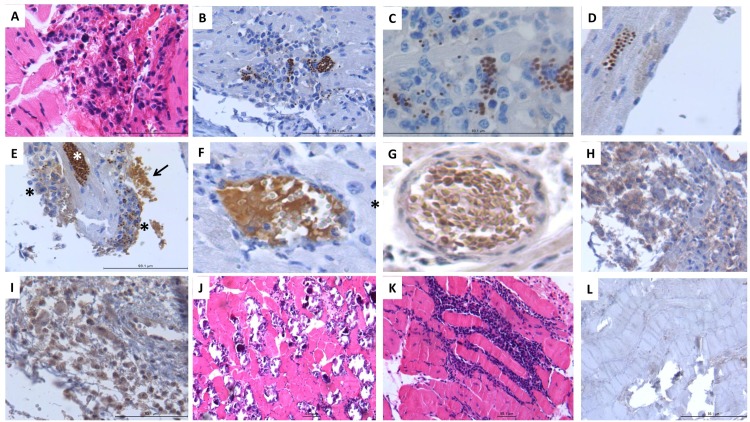
Histology and immunohistochemistry analysis of an experimental Chagas mouse model suggests that tissue-released TcHMGB can induce inflammatory cytokines production during acute infection. A) Myocardial nodule constituted by macrophages and lymphocytes in acute infection. B) These inflammatory nodules show numerous spherules with strong TcHMGB immunostaining, which correspond to *T*. *cruzi* amastigotes. C) High power micrograph show *T*. *cruzi* amastigotes immunoreactive to TcHMGB in the cytoplasm of macrophages. D) Cardiomyocytes also show in the cytoplasm numerous *T*. *cruzi* amastigotes with strong immunereactivity to TcHMGB. E) Acute myocardiopathy show heavily infected myocytes with numerous parasites showing strong immunostaining to TcHMGB (white asterisk), the endocardium shows numerous attached macrophages with phagocytosed immunostained parasites or diffuse cytoplasmic immunostaining to TcHMGB (black asterisks), fibrillar and granular material that apparently correspond to fibrin exhibited strong TcHMGB immunostaining (arrow). F) Middle size blood vessels in the myocardium show intravascular fibrin that exhibited strong TcHMGB immunostaining. G) Similar myocardial blood vessels from acute chagasic myocarditis show leukocytes with immunostaining to TNFα. H) Acute inflammatory nodules show macrophages with immunoreactivity to IL-1β. I) Numerous perivascular inflammatory cells in acute trypanosomal myocarditis show immunoreactivity to IFN-γ. J) Necrotic myocytes, fibrosis and calcification in chronic chagasic cardiopathy with extensive chronic inflammatory infiltrate (K), without TcHMGB immunostaining (L).

We finally performed a histological study on an experimental murine model of acute and chronic Chagas disease to support our observations. Small nodules of chronic inflammation, randomly distributed in the myocardium and epicardium layers (Fig 5A), characterized early cardiac lesions induced by *T*. *cruzi* in mice. These lesions showed numerous spherules that exhibited strong TcHMGB immunostaining located in the cytoplasm of macrophages or outside cells which correspond to *T*. *cruzi* amastigotes (Fig 5B). Indeed, these polyclonal rabbit antibodies were highly specific to TcHMGB permitting the identification of numerous cardiomyocytes infected with *T*. *cruzi* with minimal or absent inflammation (Fig 5C and 5D). Interestingly, some areas of the endocardium were covered by macrophages infected with strong TcHMGB-immunostained amastigotes that also showed a diffuse cytoplasmic TcHMGB immunostaining (Fig 5E, black asterisks). These infected cells were surrounded by granular or fibrilar material that also showed strong TcHMGB immunostaining (Fig 5E, arrow). These results suggest that TcHMGB protein can be released from amastigotes and can reach the cytoplasm of the infected cells as well as the extracellular space in infected tissues. Some small blood vessels also showed fibrillary or granular material that exhibited strong TcHMGB immunoreactivity (Fig 5F), which apparently corresponded to fibrin covered or associated to TcHMGB. Some monocytes or macrophages around this material inside blood vessel or around blood vessels wall, showed positive immunostaining to TNF-α, IL-1β and IFN-γ (Fig 5G, 5H and 5I). This observation is consistent with the above described results with rTcHMGB-stimulated mice, and suggest that during acute infection, *T*. *cruzi* could liberate TcHMGB, which in turn induce cytokines production. Interestingly, chronic Chagas lesions that were characterized by extensive inflammatory infiltrate, cardiomyocytes fragmentation and necrosis with calcification (Fig 5J and 5K) did not show TcHMGB staining (Fig 5L). Thus, it seems that only during early infection *T*. *cruzi* releases TcHMGB, which can contribute to induce inflammation.

## Discussion

Using *in vitro* and *in vivo* experimental systems, we demonstrated for the first time that *Tc*HMGB, the trypanosome homolog of the prototypic mammalian DAMP molecule HMGB, can be released and it is able to mediate inflammation on host cells, suggesting that the parasite´s protein may have a role in the immune response or the pathogenesis of Chagas disease.

The sequence analysis of *T*. *cruzi* HMGB highlights interesting similarities and differences with its counterpart from mammalian hosts´ and other HMGB family members (S2 Fig). HMGBs from mammals and higher eukaryotes have two HMG-box domains in tandem named “A-box” and “B-box”, followed by a negative charged domain composed of about 30 glutamate and aspartate residues usually termed “C-terminal acidic tail”. In contrast, most unicellular eukaryotes have HMGB proteins with only one HMG-box, like yeast Nhp6 (ScNhp6A, ScNhp6B) [24], *Plasmodium* HMGBs (PfHMGB-1, PfHMGB-2) [14], *Toxoplasma* HMGB (TgHMGB1a) [25] or *Entamoeba* HMGB (EhHMGB) [17]. Interestingly, *Trypanosoma cruzi* and other kinetoplastid HMGB proteins have two HMG-box domains like mice and humans (TcHMGB, LmHMGB, TbTDP1) [18,26]. The C-terminal acidic tail typical from metazoan HMGBs, which can regulate the DNA binding properties of the HMGB-box domains [27,28], is absent from most unicellular organisms included *Trypanosoma brucei* and *T*. *cruzi*, but, the putative HMGB from the related kinetoplastid *Leishmania* (LmHMGB), bears a shorter C-terminal sequence rich in serine and acidic aminoacids quite similar to that from other parasites like *Entamoeba* and *Schistosoma* (EhHMGB, SmHMGB1) [15,17,29]. Probably the most striking difference that stands out in the alignment from S2 Fig is the presence of the additional N-terminal 110 aminoacid-long sequence present in kinetoplastid HMGBs, but absent from all other HMGB family members. This sequence is highly conserved between trypanosomatids and contains a nuclear localization signal (NLS) predicted by bioinformatic methods (S2 Fig, black box) and a divergent “DEK-C terminal” DNA-binding domain that seems to be an additional point of interaction with DNA (S2 Fig, green box). Our previous results showed that besides the differences, TcHMGB keeps the DNA-architectural functions typical of the HMGBs [18].

HMGBs are known to be subjected to different post translational modifications (PTMs), which impact not only on their activities but also in their localization [30–32]. Mammalian HMGB1 has a bipartite nuclear localization signal (NLS) that directs the protein to the nucleus, where the protein plays important functions (S2 Fig, grey boxes). When macrophages are activated with the concomitant hyperacetylation of lysines present in these NLSs, the protein translocates to the cytoplasm and from there to the extracellular milieu through a non-conventional secretory pathway [21]. None of the mammalian NLSs are conserved in kinetoplastids, however, as already mentioned, trypanosomal HMGB has a putative NLS at the end of the N-terminal region specific of these organisms [18]. Results from our lab suggest that this NLS is functional and necessary to direct the protein to the nucleus, since expression of a truncated form of the protein lacking the 110 N-terminal sequence in *T*. *cruzi* epimastigotes showed the protein distributed along the cytoplasm (unpublished results). The predicted NLS also has five lysine residues that can be subjected to acetylation and probably regulate the nucleus-cytoplasm shuttle and eventual secretion, similar to what happens with HMGBs of mammals or *Schistosoma* [21,22]. Even though mutational analysis should be performed to confirm if these lysine residues are indeed acetylated to direct the protein out of the nucleus, our results show that acetylation can alter TcHMGB localization in the different life cycle stages of the parasite. Deacetylase inhibitors treatment of infected cells or free epimastigotes and trypomastigotes, which induces the hyperacetylation of most acetylable proteins, caused the relocalization of TcHMGB to the cytoplasm and an increase in TcHMGB secreted to the extracellular medium. However, the fact that TcHMGB could be detected in DACi non-treated supernatants of infected cells or free trypanosomes, suggests that other PTM or signals could be responsible for the protein release. Our immunohistochemistry results in experimental acute myocarditis showing strong TcHMGB immunostaining in intracellular amastigotes and fibrin located intravascular or deposited on the endocardium surface, suggest active expression of TcHMGB by the parasite and exportation, which could contribute to induce cytokines expression considering the positive immunostaining of pro-inflammatory cytokines by inflammatory cells near to extracellular TcHMGB deposits.

Since its first description as a late mediator of endotoxin lethality in mice by Wang *et al*.[33], our knowledge regarding HMGB1 functions as an immune mediator has increased exponentially. Human HMGB1 is released by dying cells or it can be actively secreted from immune cells like macrophages or monocytes after stimulation with lipopolysaccharide (LPS), cytokines or nitric oxide through a non-classical secretory pathway. Outside the cell, it can promote inflammation in different ways: (1) it can act as a chemotactic agent recruiting leukocytes to the site of danger, (2) it can act as a DAMP stimulating innate immune cells via pattern recognition receptors (PRR), like the Receptor for Advanced Glycation End products (RAGE) and Toll-like receptor 4 (TLR4), and (3) it can also act in association to cytokines and other molecules (LPS, lipoteichoic acid, IL-1β, chemokine CXC receptor 4 (CXCR4), RNA and DNA) binding to its major transmembrane receptors and amplifying the response to PAMPs [32,34,35].The pro-inflammatory activity of Human HMGB1 has been mapped to a highly conserved 20-aminoacid-long sequence (residues 89 to 108) located in the B-box which corresponds to the TLR4 binding site (S2 Fig, blue-box) [34,36]. Concerning trypanosomatid HMGBs, most residues in this region are either identical or conservative changes relative to metazoan HMGBs, but there are some important substitutions in this region that may produce structural changes in the protein like the three proline residues 91, 97 and 98 from human HMGB1. The RAGE-binding site, which is also critical for the HMGB1-mediated cytokine induction, is located in aminoacids 150–183 in the human protein, and it is also very conserved in metazoan HMGBs (S2 Fig, purple-box). Unlike the TLR4 binding site, this region is less conserved in other HMGB family members. In the case of *T*. *cruzi*, only four aminoacids of this region are identical to the mammalian homolog. Another outstanding difference is the substitution of cysteine 106 for a valine in *T*. *cruzi*, a serine in T. *brucei* and an alanine in *Leishmania* HMGB. These changes are surprising because cysteine 106 is very important in mammals since the protein function depends on its redox state. Three forms of human HMGB1 have been described according to the redox state of cysteine residues 23, 45 and 106 (S2 Fig, black arrowheads). When all three cysteines are in the reduced state (Thiol HMGB1), HMGB1 forms a complex with the chemokine stromal cell-derived factor (SDF-1/CXCL12) and binds to the CXCR4 receptor, increasing SDF-1/CXCL12 chemoattractant potential [37,38]. Cysteine residues 23 and 45 can form a disulphide bond, that can be accompanied by Cys106 in thiol state (disulphide HMGB1), which is the HMGB1 form that binds TLR4 leading to NFκB activation and the consequent transcription of genes involved in inflammation included those of many cytokines and chemokines [39]. Finally, the oxidized HMGB1 (cysteines oxidized to sulfonate) loss both chemoattractant and inflammatory properties and can even induce immunosuppression by the recruitment and activation of T regulatory cells [11,40,41]. These cysteine residues are conserved in mammals, birds and some worms. In contrast, none of them are present in unicellular organisms included *Plasmodium*, *Toxoplasma*, *Trypanosoma* and *Leishmania*. Besides the differences at the sequence level, particularly regarding the cysteine residues that seem to be essential for determining the final function out of the cell, both *Toxoplasma* and *Plasmodium* HMGB proteins can induce TNF-α production by macrophages *in vitro* and *in vivo* [14,25]. These observations led us to investigate the effect of TcHMGB over the inflammatory process to study its putative role on anti-trypanosome immunity. Moreover, taking into account that both host and parasite factors are thought to be responsible for the Chagas disease pathogenesis which has an important immune component, it would be expected that the parasite homolog of HMGB alarmin protein, plays a role in the disease pathogenesis. We observed that the *Tc*HMGB protein is able to activate cultured macrophages in the classical way, leading to the production of NO and the pro-inflammatory cytokines IL-1β and TNFα. The protein also induced splenocytes to produce these cytokines as well as IFN-γ, another key inflammatory mediator, after intraperitoneal administration in mice. Several parasite antigens are able to induce classical macrophage activation and the consequent increase in NO and pro-inflammatory cytokines production. This is the case of antigens like glycophosphatidylinositol-anchored mucin-like glycoproteins (GPI) [42], the TolA-like surface protein from trypomastigotes´ flagella [43] or *Tc*-52 which synergizes with IFN-γ to stimulate NO production signaling via TLR2 and conferring resistance against lethal infection in BALB/c mice [44,45]. The production of NO and pro-inflammatory cytokines like IL-1β, TNF-α and IFN-γ is critical for destroying intracellular microorganisms, included the protozoan parasite *T*. *cruzi*. However, in the case of Chagas disease, even though the parasite´s replication is controlled through pro-inflammatory cytokines and microbicidal mediators released by cells of the innate immunity and the subsequent lymphocyte subsets activated during the adaptive immunity response, not all parasites are killed and the infection persists in the host for life. There are several proposed mechanisms by which *T*. *cruzi* escapes from the immune attack to reach persistence. One of them is the poor PRR signaling because of the absence of strong PAMPs capable of activating an efficient innate immune response [1]. Kurup and Tarleton suggested that an optimal anti-pathogen immunity requires not only effective PAMPs but also the continuous expression of these PAMPs, allowing the enhancement of both innate and adaptive immune responses resulting in the final clearance of the pathogen [46]. In our study, we compared the r*Tc*HMGB stimulation effect with that of the prototypical DAMP molecule: the LPS from Gram negative bacteria. In Fig 4A and 4B, we can see that TNF-α and IL-1β gene expressions follow similar patterns in TcHMGB- and LPS-treated macrophages, although the LPS effect is stronger. This suggests that TcHMGB could be considered a putative PAMP molecule. In the *in vivo* study, although both TcHMGB and LPS induced pro-inflammatory cytokines expression, the kinetics seem to be different, what is not surprising because of the complexity of the *in vivo* model compared to the cultured cell line and the different receptors and/or cells that the two molecules could target. Our results suggest that not only structural or secreted antigens from *T*. *cruzi*, but also DAMP-like nuclear proteins, are able to induce inflammation and immune cells activation. Our hypothesis is that the trypanosome HMGB protein may be able to recognize and bind PRRs of the host, thus triggering the inflammatory response probably overlapping, at least to some extent, with the host cell DAMP molecules´ functions. This hypothesis is supported by the high conservation of the TLR4-binding region (Fig 1), although further experimental evidence is needed to confirm the protein binding to this receptor. Other PRRs could also be recognized by *Tc*HMGB contributing to the immune response. Besides the differences at the sequence level, particularly regarding the cysteine residues that seem to be essential for determining the final function out of the cell, TcHMGB, as well as its orthologs from other unicellular parasites like *Toxoplasma* and *Plasmodium*, can induce inflammatory cytokines production *in vitro* and *in vivo*. Thus, these unicellular parasites´ HMGBs may have a different way of action that might be independent of the protein redox-state, they could be influenced or regulated by different PTMs and probably they could even involve the activation of other receptors. Khan *et al*. described that the intracellular PRR TLR9, is activated by *Leishmania* and *Trypanosoma* DNA, and the inflammatory response triggered by TLR9 activation is enhanced when the parasite DNA is complexed with the host HMGB1 protein [47]. In this regard, it would be interesting to evaluate TLR9 as another candidate PRR, which could be activated by the parasite *Tc*HMGB-DNA complex. Moreover, it seems likely that DNA-bound *Tc*HMGB may have increased immunostimulatory properties, similar to *Toxoplasma gondii* homolog, where TgHMGB1a-induced TNF-α secretion by macrophages is partially dependent on the bound DNA [25]. Our previous report showing that *Tc*HMGB is a nuclear protein capable of interacting with DNA [18] is consistent with this hypothesis.

Every inflammatory response is usually counteracted by an opposite anti-inflammatory response mediated by IL-10 and TGF-β that modulates the final effect, thus preventing from an excessive inflammation that would cause severe injury and even death. Indeed, IL-10 and TGF-β play critical roles in regulation of host immune response to *T*. *cruzi*. Specific parasite molecules have shown to induce secretion of these anti-inflammatory cytokines, which have a beneficial effect for the parasite. The major *T*.*cruzi* cysteine proteinase Cruzipain (Cz), for example, induces IL-10 and TGF-β secretion as well as arginase expression by macrophages, leading to alternative macrophage activation and allowing an increased intracellular replication of the parasite [48]. We also analyzed the expression of these cytokines, after r*Tc*HMGB treatment. Interestingly, both TGF-β and IL-10 were induced by r*Tc*HMGB. This observation may seem contradictory with the pro-inflammatory properties proposed for *Tc*HMGB. However, HMGB proteins have shown to be very versatile molecules with functions ranging from chromatin architectural factors involved in transcription control, DNA replication and repair inside the cellular nucleus to a plethora of regulatory functions included cellular maturation, proliferation, motility, inflammation, survival and cell death, upon interaction with a large set of receptors out of the cell [32]. Thus, it could seem contradictory, but not surprising, the fact that TcHMGB -the parasite ortholog of HMGB1- can induce opposing effects on the immune system. As already mentioned, although human and mouse HMGB1 proteins are most frequently associated with inflammation, it is very well documented that radical changes in the protein extracellular functions occur as a consequence of PTMs and the redox state of the protein, leading not only to the lack of inflammatory induction but even to immunosuppression [11,40,41]. Indeed, some authors suggest that HMGB1´s primary role in the setting of chronic inflammation is to promote immunosuppression [49]. Anti-inflammatory or immunosuppression effects of either endogenous or therapeutically administered HMGB1 have been described in different *in vivo* systems. For example, the systemic administration of HMGB1 suppressed skin inflammation by inducing an accumulation of PDGFRα+ mesenchymal cells from bone marrow [50]. Moreover, changing effects of HMGB1 have been associated to different stages of a tuberculosis experimental model where at day 7 to 21 the oxidized HMGB1 was predominant, while during late infection only the reduced form was seen. Thus, liberated HMGB1 during experimental tuberculosis can promote or suppress the immune response and inflammation depending on its redox state [51]. Although the oxidized cysteines from HMGB1 are not conserved in *T*. *cruzi* homolog, the apparent dual effect of TcHMGB regarding its inflammatory properties, may be regulated by another kind of PTM, that deserves to be studied. Our results are solid in showing the host cells cytokine production could be influenced by the presence of a parasite HMGB protein, however, the simplification of the *in vitro* and *in vivo* models used here compared to a real Chagas disease case, could explain why TcHMGB seems to have such opposite effects. Indeed, no positive correlation between the inflammatory and anti-inflammatory cytokines expression was observed in most cases, suggesting that no double (opposite) effect is occurring in the same animal at the same time (S2 Table). Additionally, we observed that TcHMGB coincides with inflammatory TNF-α, IL-1β and IFN-γ but not with TGF-β or IL-10 in acute Chagas heart histological samples. A more detailed study of TcHMGB expression and release kinetics during *T*. *cruzi* infection would help to understand this apparent contradictory effect. Moreover, the observation that TcHMGB immunostaining was not detected in chronic heart tissues, in contrast to the strong signal in the acute samples, suggests that TcHMGB release could be differentially regulated in the different stages of Chagas Disease.

Besides the pivotal functions that TcHMGB may have, that most probably can be regulated *in vivo* by PTMs, subcellular location, parasite life cycle stage, interacting partners and disease evolution, it is important to note that TGF-β is not just an anti-inflammatory cytokine. Transforming growth factor beta is actually a pleiotropic cytokine that controls various biological processes including inflammation, fibrosis, immune suppression, cell proliferation, cell differentiation and apoptosis [52]. Regarding Chagas disease, TGF-β plays important roles at different time points from the invasion of host cells to the establishment of the chronic chagasic myocardiopathy. TGF-β secretion is activated by the parasite and is required for the invasion process. It is captured by the intracellular amastigotes and controls their proliferation and their subsequent differentiation into trypomastigotes or eventual death through apoptosis inside the host cells. Moreover, TGF-β is a key player in the development of chagasic myocardiopathy where it participates in the extracellular matrix protein production and consequent fibrosis as well as in the modulation of cardiomyocyte proliferation and death [53,54]. Fibrosis in the heart has been associated to TGF-β ability to induce expression of matrix components, to inhibit the secretion of several matrix-degrading proteases and to stimulate the synthesis of protease inhibitors. Also, extracellular matrix production by fibroblasts can be stimulated by TGF-β [55]. A correlation between increased fibrosis and increased activation of the Smad-2 pathway, through which gene expression of TGF-β-targeted genes is activated, has been documented both in Chagas patients and in an experimental model [56,57]. In this context, we can speculate that the parasite could induce *Tc*HMGB-driven TGF-β and IL-10 secretion for its own benefit interfering with macrophage microbicide activity and probably facilitating the parasite persistence, and that this cytokines production could also have a role in the chronic phase of Chagas disease.

To our knowledge, this is the first report showing that *Trypanosoma cruzi* HMGB can be released in infected tissues and act as a mediator of the immune response in mammals. The results presented herein suggest that the contribution of *Tc*HMGB to the protective immune response and immunopathology in Chagas disease should be significant and consequently it deserves to be studied in detail in the future. Moreover, *Tc*HMGB share characteristics with other HMGB family members but it also seems to have some unique features and functions, that may be conserved in other trypanosomatids, so it would be interesting to evaluate if these results could be extrapolated to other kinetoplastid-diseases like Leishmaniasis or Sleeping sickness.

## Supporting information

S1 Table MTT viability assay of RAW cells after the different treatments. (DOCX)

S2 Table Correlation analysis between inflammatory and anti-inflammatory cytokines. (DOCX)

S1 Fig Slot Blot controls. (TIF)

S2 Fig *Trypanosoma cruzi* HMGB aminoacid sequence shows the two classical HMG-box conserved motifs but has important substitutions in key residues compared to mammalian HMGB1. *Trypanosoma cruzi* HMGB protein sequence was compared to prototypical HMGB family members from unicellular and metazoan organisms and from other parasitic organisms. Sequences in the alignment are (organism, protein name, accession number): Nhp6 proteins from yeast (*Saccharomyces cerevisiae* ScNhp6A, NP_015377.1; ScNhp6B, CAA85042.1), mammalian HMGBs (*Homo sapiens* HsHMGB1, NP_001300822.1; *Mus musculus* MmHMGB1, NP_001300823.1), other metazoan HMGBs (*Gallus* GgHMGB1, NP_990233.1; *Xenopus laevis* XlHMGB1, NP_001080836.1; *Danio rerio* DrHMGB1, NP_955849.2), parasites´ HMGBs already described or related to TcHMGB (*Schistosoma mansoni* SmHMGB1, AAR85353.1*; Plasmodium falciparum* PfHMGB1, PF3D7_1202900; *Plasmodium falciparum* PfHMGB2, PF3D7_0817900; *Babesia bovis* BbHMGB-like, EDO05788.1; *Toxoplasma gondii* TgHMGB1a, TGME49_210408; *Entamoeba histolytica* EhHMGB, XP_657292.1; *Leishmania major* LmHMGB, LmjF.29.0850; *Trypanosoma brucei* TbHMGB1 (TbTDP1), Tb927.3.3490; *Trypanosoma cruzi* TcHMGB, TcCLB.507951.114).
(TIF)
