# Supplementary material for: Trypanosoma cruzi High Mobility Group B (TcHMGB) can act as an inflammatory mediator on mammalian cells
Source: PLoS Negl Trop Dis. 2017 Feb 8;11(2):e0005350. doi: 10.1371/journal.pntd.0005350 (PMC5319819; doi:10.1371/journal.pntd.0005350)
Supplement: S1 Table — (DOCX) [file pntd.0005350.s001.docx]

| **OD (530nm)** | **Mean** | **SD** |
| --- | --- | --- |
| **Control** | 0.77 | 0.2 |
| **rGST** | 0.88 | 0.08 |
| **rTcHMGB** | 0.94 | 0.25 |
| **LPS** | 0.9 | 0.02 |

**S1 Table: MTT viability assay of RAW cells**
