# Supplementary material for: Trypanosoma cruzi High Mobility Group B (TcHMGB) can act as an inflammatory mediator on mammalian cells
Source: PLoS Negl Trop Dis. 2017 Feb 8;11(2):e0005350. doi: 10.1371/journal.pntd.0005350 (PMC5319819; doi:10.1371/journal.pntd.0005350)
Supplement: S2 Table — (DOCX) [file pntd.0005350.s002.docx]

| **Correlations** | **TNF-a** | **IL-1b** | **IFN-g** |
| --- | --- | --- | --- |
| **IL-10** | rS: -0.5638  P=0.0148 | - | - |
| **TGF-b** | - | - | rS: 0.65  P=0.0078 |

S2 Table. Correlation analysis of the expression of pro and anti-inflammatory cytokines measured in sera from mice treated with Tc-HMG.

Correlation analyses were carried out using the non parametric Spearman test (rS). The GraphPad Instat 6.0 software (GraphPad, California, USA) was applied for graphs and statistical analysis. Differences were considered significant when P value was <0.05.
