# Supplementary figures and images for: Trypanosoma cruzi High Mobility Group B (TcHMGB) can act as an inflammatory mediator on mammalian cells
Source: PLoS Negl Trop Dis. 2017 Feb 8;11(2):e0005350. doi: 10.1371/journal.pntd.0005350 (PMC5319819; doi:10.1371/journal.pntd.0005350)

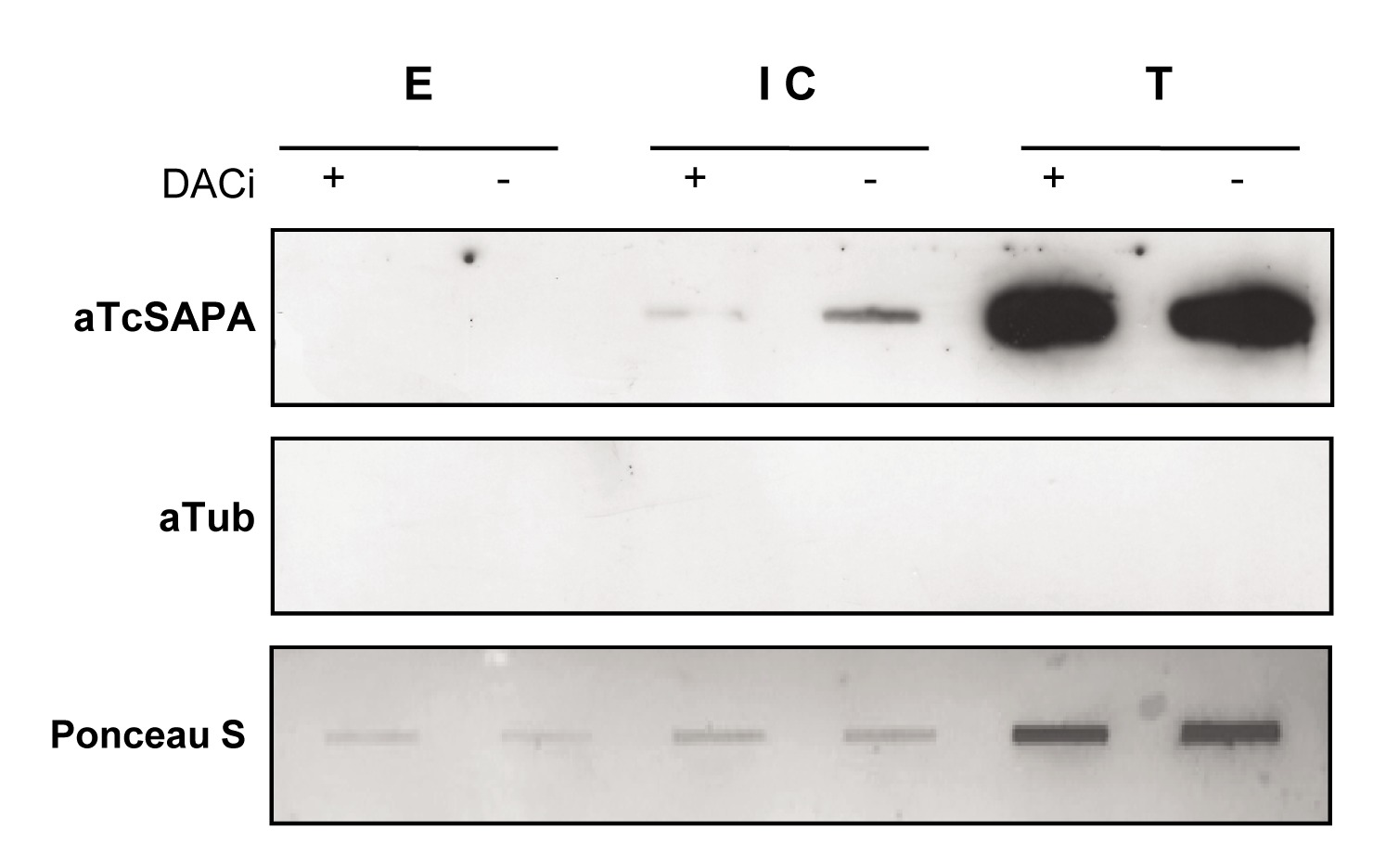

Supplement: S1 Fig — (TIF) [file pntd.0005350.s003.tif]

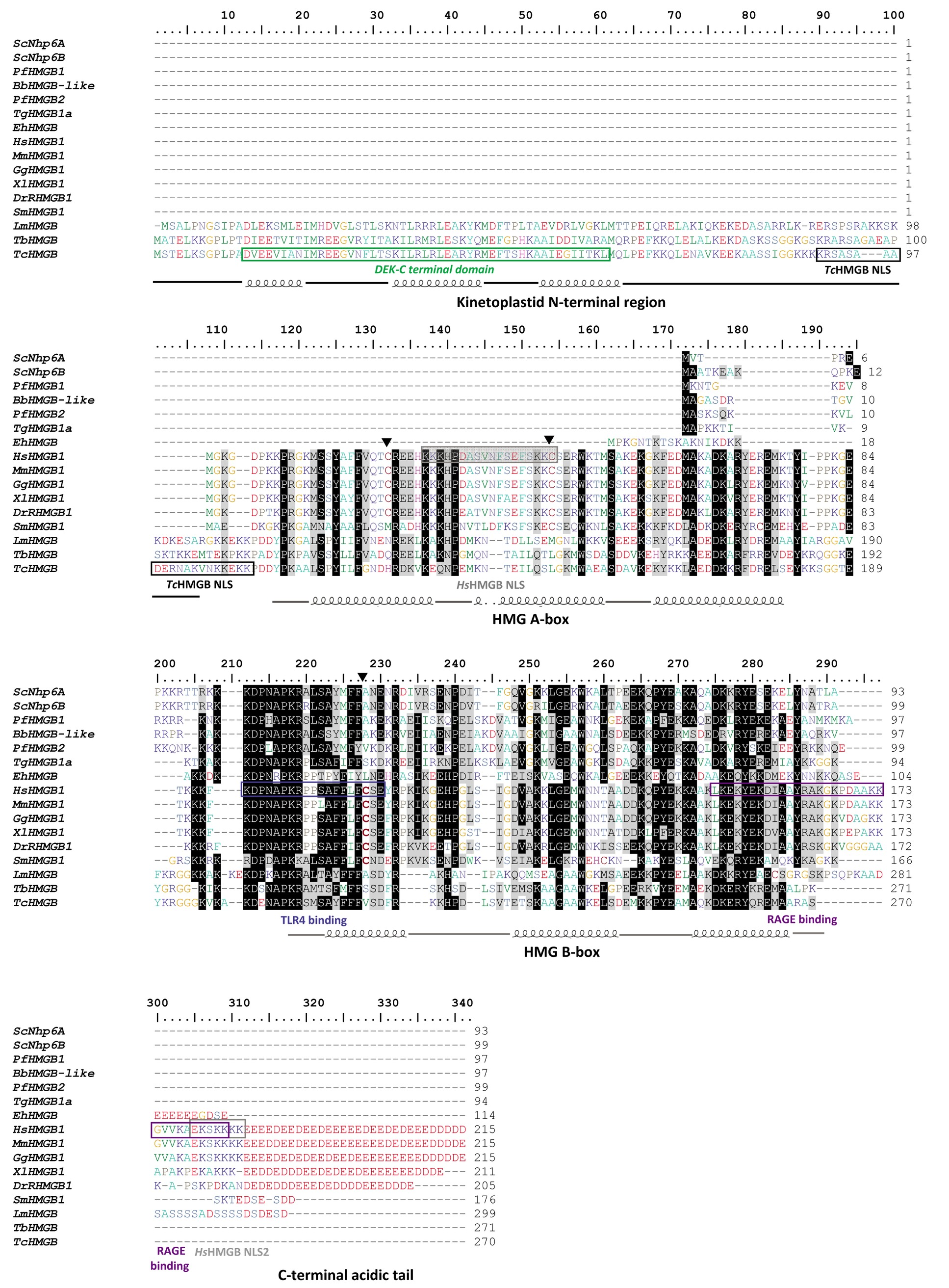

Supplement: S2 Fig — Trypanosoma cruzi HMGB protein sequence was compared to prototypical HMGB family members from unicellular and metazoan organisms and from other parasitic organisms. Sequences in the alignment are (organism, protein name, accession number): Nhp6 proteins from yeast (Saccharomyces cerevisiae ScNhp6A, NP_015377.1; ScNhp6B, CAA85042.1), mammalian HMGBs (Homo sapiens HsHMGB1, NP_001300822.1; Mus musculus MmHMGB1, NP_001300823.1), other metazoan HMGBs (Gallus GgHMGB1, NP_990233.1; Xenopus laevis XlHMGB1, NP_001080836.1; Danio rerio DrHMGB1, NP_955849.2), parasites´ HMGBs already described or related to TcHMGB (Schistosoma mansoni SmHMGB1, AAR85353.1; Plasmodium falciparum PfHMGB1, PF3D7_1202900; Plasmodium falciparum PfHMGB2, PF3D7_0817900; Babesia bovis BbHMGB-like, EDO05788.1; Toxoplasma gondii TgHMGB1a, TGME49_210408; Entamoeba histolytica EhHMGB, XP_657292.1; Leishmania major LmHMGB, LmjF.29.0850; Trypanosoma brucei TbHMGB1 (TbTDP1), Tb927.3.3490; Trypanosoma cruzi TcHMGB, TcCLB.507951.114). (TIF) [file pntd.0005350.s004.tif]
